# The macrostructure of narratives produced by children acquiring Finnish Sign Language

**DOI:** 10.1093/jdsade/enae049

**Published:** 2024-11-14

**Authors:** Heta Pietarinen, Laura Kanto

**Affiliations:** Department of Language and Communication Studies, University of Jyväskylä, Seminaarinkatu 15, PO Box 35, FI-40014, Jyväskylä, Finland; Department of Language and Communication Studies, University of Jyväskylä, Seminaarinkatu 15, PO Box 35, FI-40014, Jyväskylä, Finland

**Keywords:** assessment and testing, bimodal, bilingualism, development, sign language

## Abstract

This article investigates the narrative skills of children acquiring Finnish Sign Language (FinSL). Producing a narrative requires vocabulary, the ability to form sentences, and cognitive skills to construct actions in a logical order for the recipient to understand the story. Research has shown that narrative skills are an excellent way of observing a child’s language skills, for they reflect both grammatical language skills and the ability to use the language in situationally appropriate ways. This study was conducted using the FinSL Narrative Skills Production Test assessment to observe how narrative skills develop in children between the ages of 4 and 11 who acquire FinSL in their natural language environments. The results show that the narrative skills of children acquiring FinSL develop following the same guidelines found in other signed and spoken languages. Narrative structure and content increase with age.

Narrative skills are a broad reflection of language development. The development of narrative skills usually proceeds in stages, starting in the prelinguistic stage (Suvanto & Mäkinen, 2011) and continuing into early adulthood (Loukusa et al., 2020). The development of storytelling skills is based on the prelinguistic stage, when children develop their skills through interacting with their environment and with adults (Suvanto & Mäkinen, 2011). Through these interactions, when a child does not have any specific developmental challenges, the child’s narrative skills develop at the same pace as other social and linguistic cognitive skills (Loukusa et al., 2020).

Narrative tasks are used widely for studying children’s language skills. Narratives allow the assessment of different elements of language in a relatively short language sample. When studying and assessing the development and characteristics of children’s storytelling skills, the narratives produced by children can be viewed in different ways. Narratives can be divided into micro- (e.g., lexicon, grammar, and syntax) and macrostructures (e.g., story structure, organization, and coherence), and, therefore, narratives provide an opportunity to effectively examine children’s language use and narrative skills from a variety of perspectives. Narrative requires children to master a wide range of linguistic and cognitive skills and to be able to coordinate different skills in producing a narrative. Composing a narrative requires vocabulary and the ability to form sentences that are understandable. A narrator must be able to conduct a story that has logical references, control the relations between cause and effect, and understand what information the recipients hold and what they do not (Suvanto & Mäkinen, 2011). Therefore, narrative skills testing is essential when looking at children’s language development from research, clinical, and pedagogical perspectives.

Although the study of children’s narrative skills provides a valuable source of information on child development, research on the narrative skills of children acquiring sign language in general has been rather limited. In the context of Finnish Sign Language (FinSL), there has been very little research on children’s FinSL development to provide a reliable overall picture of the development of children’s language skills. Previous research on language development in children acquiring FinSL has mainly focused on the vocabulary and structural parts of the language of children under the age of 5 (Kanto, 2016; Kanto et al., 2021; Takkinen, 2003, 2013), and narrative skills have hardly been studied at all. In recent years, the field of sign language research has sought to develop standardized methods for assessing language development.

The aim of this research is to study the narrative skills of children acquiring FinSL. In order to make the first opening for the research on the narrative skills of children acquiring FinSL, this article focuses on the study of the narratives produced by children in a broader sense, with particular attention to the macrostructure and structural complexity of the narratives. The narratives produced by the children studied were elicited with the FinSL Narrative Skills Production Test (FinSL-PT) (Kanto & Syrjälä, 2019). The FinSL-PT assessment tool was adapted from the British Sign Language Production Test (BSL-PT) and allows the assessment of children’s narrative skills in FinSL. The BSL-PT (Herman et al., 2004) was developed for British Sign Language and is one of the first assessment tools developed for sign language to measure children’s narrative skills.

## The macrostructure of narratives

**Figure 1:**
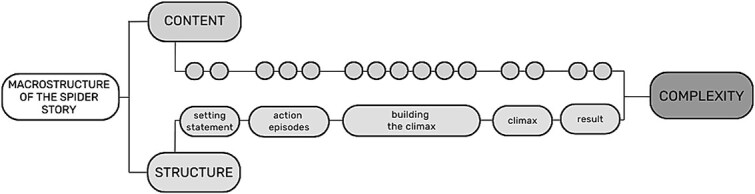
The macrostructure of the spider story in the FinSL-PT.

A story is a linguistic description of a series of events that can be based either on real-life events or on imagination. A story consists of individual expressions that are linked together cohesively. A coherent, well-structured story is easy and effortless for the recipient to understand (Loukusa et al., 2020). When analyzing produced stories more closely, narratives can be divided into microstructure and macrostructure. The microstructure includes the linguistic level of the narrative (e.g., vocabulary, grammar, and syntax), while the macrostructure (see Figure 1) includes the overall organization of the story (e.g., the structure of the story, story content, and coherence) (Kunnari et al., 2016).

One of the most commonly used approaches to analyzing the structure of narratives is the story grammar model developed by Stein and Glenn (1979), in which a story is divided into a setting statement and one or more episodes. The setting statement includes the introduction of the main characters, and it describes the social and physical context of the story. An episode is a behavioral sequence that can be broken down into specific content units, including external and/or internal events that affect a character, the character’s internal response, the character’s external response, and the consequences of the character’s response (see Figure 1). According to the story grammar model, in the simplest story there is only the setting statement and one episode, but most stories are more complex and have two or more episodes that can be related in different ways (Stein & Glenn, 1979).

The macrostructure can be used to analyze a story’s complexity (i.e., how many details and different structure episodes are found in the story). There are several different ways to determine the structural complexity of a story, including the number of details in the episodes, the number of complete/incomplete episodes (a complete episode being an initiating event, attempt, and consequence), or the total number of episodes found in a story (Kunnari et al., 2016).

Figure 1 represents the macrostructure of the spider story that was originally used in the BSL-PT. The spider story is a story based on a video in which two children act through a series of events without communicating in any signed or spoken language. The story plot is a sequence of repeating similar events with a thrilling climax and consequences. The plot is designed to elicit children’s FinSL narratives for assessing important structures of narratives and the production of different sign language grammatical elements (Herman et al., 2004). The story is divided into five different episodes. For each episode, there is a specific number of content units that represent the detailed description of different events in the story represented in Figure 1. By analyzing the features and numbers of content units and episodes, the complexity of children’s produced narratives can be explored.

## The development of narrative skills

Before the age of 3, children’s narrations are typically short, one- or two-sentence descriptions of events that happened to the child in the past (Stadler & Ward, 2005; Suvanto & Mäkinen, 2011). By the age of 3–4, children have acquired sufficient linguistic and cognitive skills to expand their narratives beyond situationally specific narration (Loukusa et al., 2020) and begin to produce their first stories (Stadler & Ward, 2005). When narrating from a picture, a 3-year-old is still only describing single events (Mäkinen & Kunnari, 2009) and cannot yet form a coherent, complete story. By the age of 4, narrative skills have developed to the point at which a child can produce a story in a picture-based narrative task, although main points and other relevant elements may still be missing (Suvanto & Mäkinen, 2011).

At the age of 4–5, children can already tell stories about their own experiences, as well as about pictures, but the stories are often still vague and lack logical structure (Morgan, 2005). When retelling stories, children at this age better remember the surprising events where the story differed from what was expected to happen than the events that unfold in a normal way without any unusual details (Bruner, 1990). However, there is evidence showing that between the ages 4 and 5, there seems to be significant development in using story grammar units (Muñoz et al., 2003; Price et al., 2006), and the structural complexity of the stories increases. Whereas the stories of 4-year-olds are still narrow and may lack many structural elements, by the age of 5, stories already contain the essential elements. However, it is still difficult for children to express causality, and their stories may lack conclusions (Mäkinen & Kunnari, 2009).

Although children of this age have already mastered the internal logic of episodes, at the age of 5–6 years, the ambiguity of references is still typical, especially in fictional narratives. For this reason, the listener may still find it difficult to understand the narrative (Loukusa et al., 2020). However, at this age, children are able to narrate coherent stories, including all the episodes (Suvanto & Mäkinen, 2011). The length and details of a child’s stories increase as well. A 6-year-old has developed important skills for fluent narration, such as theory of mind (Morgan, 2005). Theory of mind is the ability to understand that other people may feel and experience things differently from the child (Stadler & Ward, 2005). The development of theory of mind helps the child to understand and explain different elements of the story, such as the cause and effects of the story and characters’ goals, intentions, and emotions.

Seven-year-old children’s narratives have clear narrative structure elements, and they reflect narrative structure rather than only describe events (Suvanto & Mäkinen, 2011). From the age of 4 until the age of 7, narrative skills can develop quite rapidly, by leaps and bounds. Schneider et al. (2006) found that narrative structure development starts to slow down at the age of seven. Still, the other language skills affecting narrative skills continue to develop throughout adolescence (Morgan, 2005) and into early adulthood (Loukusa et al., 2020).

Although there has been a significant amount of research on narrative development in children acquiring spoken languages, the research on the narrative skills of children acquiring signed languages has globally been quite limited. Previous research conducted in signed languages focusing on children’s development of narrative skills, specifically concerning the macrostructure, shows that the development is very similar to what has been found in spoken languages. Studies done by Reilly (2001) and Morgan (2005) with deaf, natively signing children’s narratives, and in a study by Marschark et al. (1994) with deaf children with hearing parents, all found that the episode structure was age-appropriate compared to what is known from earlier studies done in spoken languages. Vercaigne-Ménard et al. (2001) studied the early narrative development of two deaf children, analyzing the macrostructure of their stories at the age of 4 and again at the age of 6. They found that the episode structure of the stories at the age of 4 was similar to hearing 2-year-olds’ stories, but the deaf children caught up in development by the age of 6. In a study done by Becker (2009) in German sign language, the episode structure of deaf children was found not to be adequate to their age when compared to their hearing peers. This study was done using pictures to elicit stories from 8-to-17-year-olds. However, when studying narrative development with the assessment material adapted from the BSL-PT to German sign language, Becker (2009) found no difference between the deaf and hearing children’s episode structures in their signed stories elicited from video material (Enns et al., 2021). In Becker’s study, the children were either native signers or had access to sign language at the latest from daycare.

## Assessing children’s narrative skills

As explained above, children’s narrative skills require and overlap on a large scale of different skills they must acquire and develop. For this reason, the assessment of children’s narrative skills is a traditional and important part of child language assessment. However, the assessment of narrative skills can be carried out in different ways, and the choice of assessment tool or elicitation method depends on the age of the child and the language being assessed, as well as the aspect of narrative skills or child development that is to be examined (see, e.g., Crawshaw et al., 2020; Haug et al., 2018). Narrative tasks can be a spontaneous situation with free play, or they can be elicited by using picture or video materials representing an example story. Spontaneous speech is more difficult to analyze; comparing children is harder, not just because their stories may differ significantly but also because a free situation does not necessarily stimulate children in the same way to produce a story (Mäkinen & Kunnari, 2009). By using specific elicitation material and methods, children’s narratives can be collected and analyzed in more systematic ways, and in this manner children’s results can be compared with each other and with their age peers more easily.

However, previous studies have observed that the use of different elicitation materials and methods may result in very different features in children’s narrative skills, even among the same children. For example, it has been suggested that using video-based elicitation materials to assess children’s narrative skills might benefit and support retelling compared with storybook- or still-image-based materials. Video-based materials might engage children strongly to conduct the task, increase understanding of the story, and activate different areas of cognitive processing, resulting in stronger mental encoding and better recall from that of still-image tasks (Crawshaw et al., 2020; Diehm et al., 2020; Puupponen & Kanto, n.d.). Given the wide range of skills required of children to narrate a story and the many ways in which narratives can be elicited, a thorough assessment of children’s narrative skills is methodologically challenging, which might also influence the validity and generalizability of the results of research or language assessments.

Developing narrative assessment tools for children is always a demanding task. However, there are a few specific challenges in the context of narrative assessments of children acquiring sign language. Assessing children’s signed narratives always requires video recordings of the narratives that can be analyzed afterward. This requires high-quality recording equipment and analysis tools. Additionally, the analysis of video recordings is time-consuming and requires specific expertise, even from test administrators who are fluent in sign language (Herman & Rowley, 2022). Sign language research in early stages, small sample sizes and heterogeneity in populations of children acquiring sign language, and available research resources also challenge the development of tools for children’s sign language assessment in general.

Partly for these reasons, only a few assessment tools for different sign languages have so far been developed to assess the narrative skills of children acquiring sign language (Herman et al., 2004; Hermans et al., 2010; Maller et al., 1999). However, the development of assessment tools to evaluate children’s sign language skills is highly important for a variety of purposes, for example, to track sign language development, plan educational and interventional practices, and follow up with language learning. For these reasons, research on children acquiring sign language, especially in terms of developing assessment tools and studying their various features, is sorely needed in this particular research field. The latest research on the test adaptation processes, in which the developed test is adapted from sign language to another sign language and the adaptation processes and their positive outcomes are documented in detail, has encouraged researchers to consider test adaptation to increase the availability of sign language tests for a variety of different sign languages (Enns et al., 2021; Enns & Herman, 2011; Kanto et al., 2021; Mann et al., 2016). This study investigates the characteristics of children’s narrative skills in FinSL using the FinSL-PT adapted from the BSL-PT, and in doing so it also aims to address the important and central needs of the research field as discussed above.

## The present study

The focus of this study is especially on the features of content and structure in narratives produced by children between the ages of 4 and 11 years old who are acquiring FinSL. More specifically, this study examines how different content units are incorporated in the story structure of narratives produced by children at different ages. The aim of this study is to examine if there is a correlation between age and the production of the macrolevel elements of narratives by analyzing children’s scores on the FinSL-PT for narrative structure and content. Narrative structure and content are analyzed quantitatively using the scores obtained using the assessment tool. Structural complexity is analyzed by determining how many episodes children produce and whether there is a pattern in which the order of different episodes appears in stories as children grow older. The primary research questions are as follows.

(1) Is there a correlation between age and the production of the narrative structure and content?
(2) What episodes of narratives do children in different age groups produce, and does the complexity of the narratives increase with age?

## Methods

### The data

The data comprise video-recorded narratives by 94 children ages 4–11 (see Table 1). Altogether, 42 children were deaf, and 52 children were hearing. Of the deaf children, 26 had at least 1 deaf parent, and 16 had hearing parents. 23 of the deaf children had FinSL as the official language used in their education. Of the hearing children, 49 had at least 1 deaf parent, and 3 had 2 hearing parents. Two of the hearing children had FinSL as the official language used in their education, both of whom had deaf parents. The children were recruited for the study by informing signing families of the research via social media, school or day care personnel, and sign language associations. All willing children and families were included in the study, regardless of the hearing status of the child or parents. The only criteria for participating in the study was that the child acquire FinSL from their linguistic environment and be between 4 and 11 years old. Detailed background information and features of children’s linguistic environments were acquired using a parental questionnaire. All the children lived in bi- or multilingual environments. The children were from different parts of Finland.

**Table 1:** The distribution of children divided by age and hearing status of the child and their parents.

	Deaf child of deaf adult	Deaf child of hearing adult	Hearing child of deaf adult	Hearing child of hearing adult
4 yo (*N* = 9)	2	0	7	0
5 yo (*N* = 10)	3	2	5	0
6 yo (*N* = 10)	1	1	7	1
7 yo (*N* = 18)	6	4	8	0
8 yo (*N* = 12)	3	4	5	0
9 yo (*N* = 13)	4	2	6	1
10 yo (*N* = 9)	2	0	6	1
11 yo (*N* = 13)	5	3	5	0
All (*N* = 94)	26	16	49	3

The total duration of the data was 3 hr and 59 min. The total duration of the stories produced by the children was 1 hr and 56 min (*M* = 1 min, 19 s, *SD* = 44.96 s, min = 1.4 s, max = 4 min, 51 s).

### Data collection

The data were collected by using the FinSL-PT (Kanto & Syrjälä, 2019). The FinSL-PT is an assessment tool designed to measure the narrative skills of children who acquire FinSL in their natural environments. It was adapted from the BSL-PT (Herman et al., 2004), which is one of the first assessment tools designed to assess narrative skills for signed languages. Previously, the BSL-PT was adapted also for American Sign Language (ASL) and German Sign Language (DGS). When the BSL-PT was adapted for ASL, the research team refilmed the spider story to better reflect different cultural features (Enns et al., 2021). During the adaptation process of the FinSL-PT, the spider story from the ASL Expressive Skills Test (Enns et al., 2019) was found to be culturally more suitable compared to the original spider story video from the BSL-PT, and for this reason it was chosen for the elicitation material in the FinSL-PT used in this study.

The FinSL-PT is a narration task that has two parts. In the first part, the children are asked to watch a 3-min-long, language-free video (the spider story) and then to tell the story to a test administrator who has not seen the video. In the second part, the children are asked three questions concerning the events of the story to ensure comprehension of the story (Kanto & Syrjälä, 2019). The whole situation is video recorded for later analysis. The story in the video features two children acting through a series of events without communicating in any signed or spoken language. The video does not provide any linguistic examples for the children. The story plot is a sequence of repeating, similar events with a thrilling climax and consequences. The plot is designed to elicit children’s signed narratives for assessing important structures of narratives and sign language grammar.

The assessments were carried out by multiple assessors. All the assessors were trained to use the assessment tool. All assessors had fluent skills in FinSL and were both deaf and hearing. The assessment sessions were conducted in FinSL.

At the beginning of the assessment session, the assessor would instruct the child to first watch a video that the assessor would not see. Then, the child should tell the assessor what happened in the video as accurately as possible, and after that, the assessor would ask the child three questions related to the events in the video. The assessor would also tell the child that there was a camera filming the whole situation.

The assessors were asked to place the camera so that both the child and the assessor would appear in the video. However, there were a few videos in which the assessor was not in the picture or was facing away from the camera so that it was not clear what the assessor was signing. The children were advised to sit so that their faces were turned slightly toward the camera and not directly toward the assessor. This was not possible in the assessment sessions that were carried out via remote connections. In these remotely conducted sessions, the assessor advised the child to set the camera so that the video would not only show their faces but also their torsos so that they could see what they were signing.

The assessor was allowed to encourage the children during the narration with minimal normal interactions, such as nodding, smiling, or using certain signs, such as YES. If the child seemed to stop in the middle of the story, the assessor was allowed to encourage the child once by asking, for example, “What happened next?”

After the child was finished with their story, the assessor would ask them three questions related to the video. Children were asked to list things they saw on the video and to give reasons for the actions of the characters on the video (qn 1. TRAY WHAT ON, qn 2. WHY BOY THROW SPIDER, qn 3. WHY GIRL TEASE BOY). Examples of the questions were prerecorded, and the assessor was advised to ask the questions the same exact way in every assessment session.

The narratives were collected as a part of a larger research project at the University of Jyväskylä (Kanto & Syrjälä, 2024). 24 of the narratives were collected in 2018 as a part of the pilot study, and the remaining 70 narratives were collected between 2019 and 2022. All the data were collected in the children’s own environments: at home, at day care centers, or at schools. Because of the Covid-19 pandemic, 29 of the assessments were completed via remote connections.

### Annotation

All the videos were annotated and scored according to the scoring sheet included in the FinSL-PT. The scoring of children’s narratives allowed for statistical comparisons of narrative skills. The annotation and scoring of the test included three parts: narrative content (15 narrative episodes, responses to questions); narrative structure (setting statement, action descriptions, climax building, climax, resolution, conclusion); and FinSL grammar (descriptive gestures, demonstrative verbals, aspectual modification, modification of manner, constructed action). In this research, we focus on the narrative content and structure of the stories produced by the children studied.

The stories were annotated using ELAN (Eudico Linguistic Annotator; Crasborn & Sloetjes, 2008) by two annotators. Both the deaf and hearing annotators were fluent in FinSL. Annotators worked separately, but in unclear cases, the annotators consulted together to find suitable solutions and keep the annotations consistent. The annotations were done partly based on the scoring sheet of the FinSL-PT and using separate rows for different factors influencing the assessment: right hand, left hand, observations, narrative structure, narrative content, FinSL structure, and total duration (see Table 2).

**Table 2:** ELAN (Eudico Linguistic Annotator) tiers used in the annotation of stories.

Tier name	Description
Right hand	A gloss or depiction indentifying the sign in Finnish, undentified signs marked ZZZ
Left hand	A gloss or depiction indentifying the sign in Finnish, undentified signs marked ZZZ
Observations	Pointing signs, depicting signs, non-manual actions, note of uncertain glosses
Narrative content	Content units, answers to the questions
Narrative structure	Episodes (Setting statement, Action, Building climax, Climax, Resolution)
FinSL structure	Spatial verbs, agreement verbs, aspect, manner, and role shift
Total duration	Duration the whole continuous stretch of narration

The right hand and left hand rows contained annotations of all the manual movements of the hands using glosses or depictions of the hands’ movements. In signed languages, non-manual components are particularly important. In narration, non-manual behavior (e.g., facial expressions or mouthed words) were annotated in the observations row. In the narrative content annotation row, story content units were annotated according to the FinSL-PT. The units consisted of single events, such as “boy watches TV” or “girl sees a spider.” The specific signs used to produce the content unit were irrelevant, and a sentence with the corresponding meaning was approved. The correct answers to the questions asked after the retell were also annotated in the narrative content row.

### Scoring

After annotating the stories, they were scored using the FinSL-PT guidelines. The content of the narratives was assessed in terms of the amount of information produced by the child. The scoring of narrative content was broken down by episodes, as the content score is related to the structure of the narrative. Scoring for narrative content does not require the child to express the content unit using specific signs; what is essential is that the information is expressed in a way that can be understood. Moreover, the child does not need to produce every detail of the story, provided that through cohesion and references, the content can be understood without the recipient knowing the story in advance (Kanto & Syrjälä, 2019). In addition to the episodic content, the children received one point for the narrative content if they provided additional information for the story, for example, by describing the character’s point of view or by expressing the child’s own opinion that was not directly related to the content of the story but provided additional information.

The narrative structure scores were linked to the content points. Narrative structure was assessed by episodes, and for each episode produced by a child, points were awarded according to the score the child had received for the content in the episode. In addition to the five episodes found in the story, a child received one point if the story was told in the correct chronological order and one point if the child expressed their own opinions about the characters in the story, their emotions, or their characteristics during the story (Kanto & Syrjälä, 2019). For the setting statement, the child received one point for each reference to the characters in the story. After the setting statement, the story includes a series of actions, and the child was given a point for each description of an action. In the climax building, climax, and resolution, the child received one point for each reference to the events of those episodes (Kanto & Syrjälä, 2019).

To evaluate the reliability of the scoring, two annotators independently scored twenty narrations (21%) produced by the children. For the analysis, at least two videos were randomly selected from each age group for the inter-rater agreement analysis. The agreement regarding the scoring between two annotators was 93% on narrative scoring and 95% on narrative structure scoring. Additionally, to measure agreement, Cohen’s Kappa and the correlation coefficient Pearson’s *r* were estimated. Following the guidelines provided by Landis and Koch (1977), the agreement of judgments for scoring the narrative content (*k* = 0.85) and for scoring the narrative structure (*k* = 0.93) was very good. Additionally, Pearson correlation resulted in a highly significant correlation between the two coders in narrative content at 0.96 (*p* < .01) and in narrative structure at 0.96 (*p* < .01), indicating that inter-rater reliability was very good.

### Statistical analyses

The scoring of the narratives allowed the results to be analyzed using quantitative statistical methods. The results of total score, content score, and structure score were analyzed in SPSS via the Kruskal–Wallis test for statistically significant differences between groups. The *p*-values were adjusted for multiple comparisons with Bonferroni correction. The variables were explored for outliers using the *z*-score, and there were no outliers. Spearman’s correlation was used to measure the correlation between age and the results. The complexity of the stories was analyzed by examining the mean scores of the children in every age group within the episodes.

## Results

**Figure 2:**
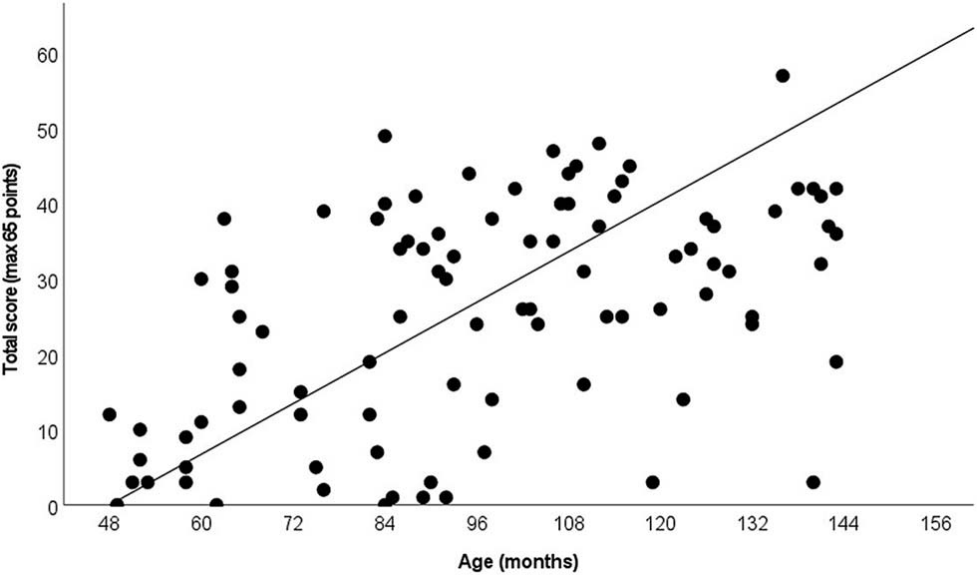
The total scores of children in the FinSL Narrative Skills Production Test (*r* = 0.494, *p* < .001)

The analysis of the results of the FinSL-PT shows that there is a correlation between age and narrative skills. As age increases, the total scores of the children in the test increase. Figure 2 and Table 3 show the total scores of the children in the FinSL-PT. The total scores include narrative structure, narrative content, answers to the questions, and FinSL scoring. Throughout the age groups, the variation is wide (see Table 3). Follow-up analyses revealed specific differences between 4-year-old children and 8- (*p* = .002), 9- (*p* = .001), and 11- (*p* = .001) year-olds.

**Table 3:** The mean scores of age groups.

	Total score			Story content			Story structure		
	Mean	*SD*	Min-Max	Mean	*SD*	Min-Max	Mean	*SD*	Min-Max
4 yo (*N* = 9)	5,670	3,937	0–12	2,560	1,667	0–5	1,670	1,803	0–5
5 yo (*N* = 10)	21,800	11,361	0–38	10,000	4,989	0–15	6,800	3,048	0–10
6 yo (*N* = 10)	18,700	14,392	2–39	7,500	5,622	1–17	4,700	3,466	0–11
7 yo (*N* = 18)	25,220	16,879	0–49	11,000	6,886	0–19	6,170	4,218	0–12
8 yo (*N* = 12)	29,830	11,816	7–47	12,920	4,776	3–18	7,500	3,119	1–11
9 yo (*N* = 13)	34,080	13,444	3–48	14,920	4,555	3–19	8,310	3,093	0–11
10 yo (*N* = 9)	30,330	7,228	14–38	15,110	3,180	7–18	8,000	1,732	4–10
11 yo (*N* = 13)	33,770	13,473	3–57	14,620	5,075	1–21	8,380	2,501	1–11
All (*N* = 94)	25,780	14,922	0–57	11,390	6,180	0–21	6,600	3,602	0–12
	(*H* (7) = 26.59, *p* < .001)			(*H* (7) = 31.85, *p* < .001)			(*H* (7) = 24.67, *p* < .001)		

### Narrative content and structure results

**Figure 3:**
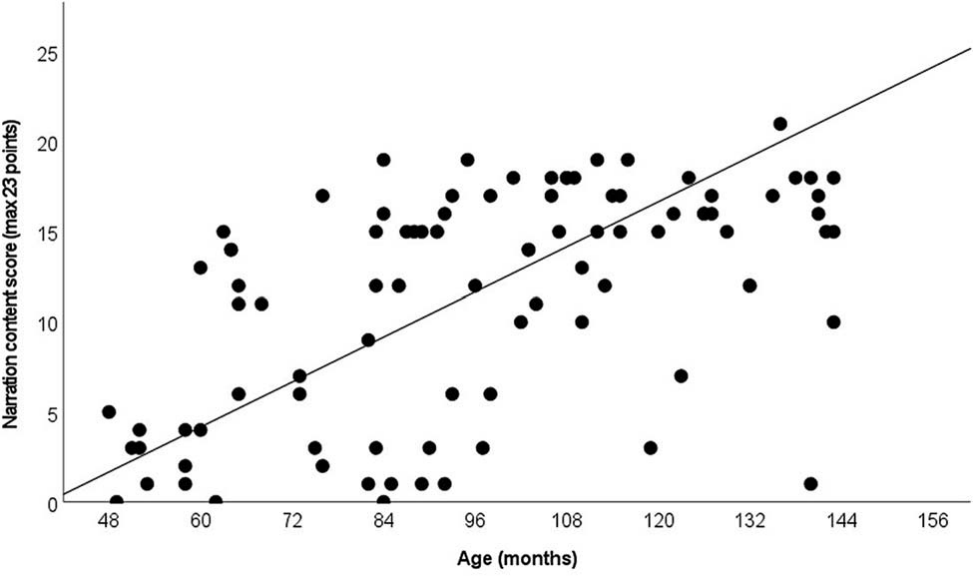
The scores of children in story content (*r* = 0.541, *p* < .001)

The first research question addresses the correlation of the results in narrative content and structure with age. The total scores for content include the points children received by answering the three questions they were asked after they had told the story to the assessor. Figure 3 shows the results of the story content analysis. There is a strong correlation between age and the results (*r* = .541, *p* < .001). Follow-up analyses revealed specific differences between 4-year-old children and 8- (*p* = .039), 9- (*p* = .000), 10- (*p* = .002), and 11- (*p* = .001) year-olds.

**Figure 4:**
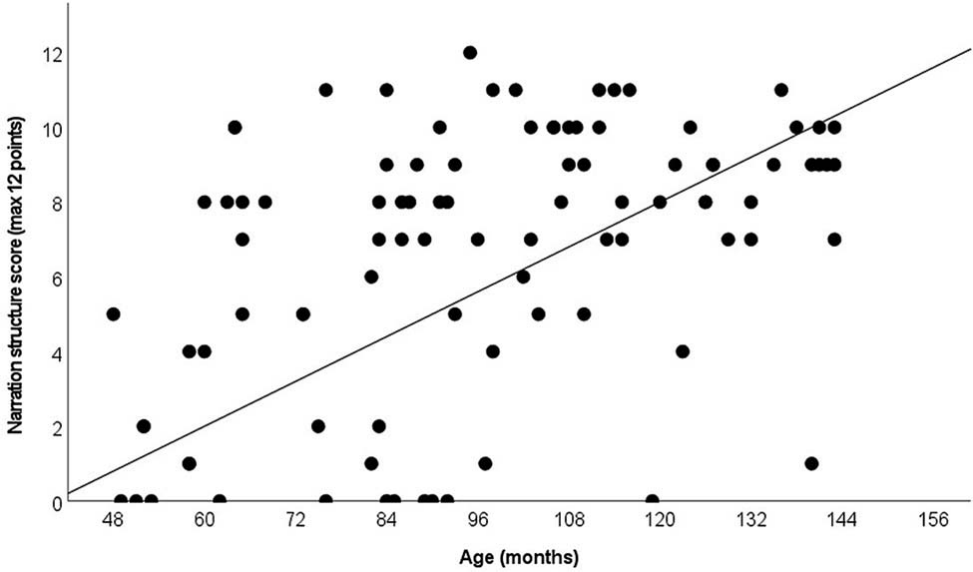
The scores of children in story structure (*r* = 0.452, *p* < .001)

When analyzing the structure (see Figure 4) of the narratives separately, a strong correlation is observed with age (*r* = .452, *p* < .001). Follow-up analyses revealed specific differences between 4-year-old children and 8- (*p* = .033), 9- (*p* = .002), 10- (*p* = .047), and 11- (*p* = .002) year-olds.

### Narrative complexity results

The second research question focused on the complexity of the story. The aim was to find out which episodes were likely to be included in the stories at which ages, as well as how much children of different ages included content units in the episodes.

**Figure 5:**
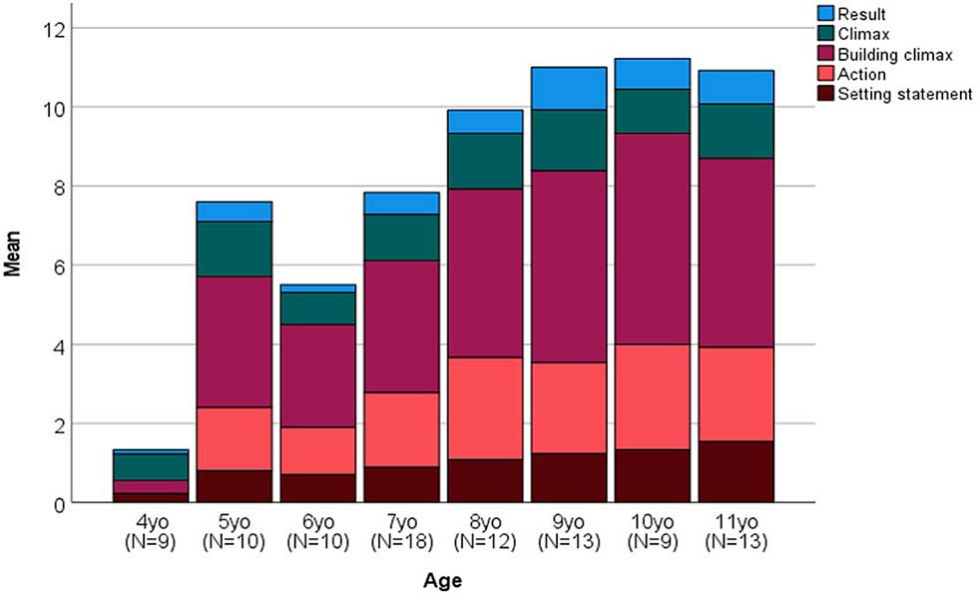
Age group complexity of the narratives by mean score of content in each episode.

Figure 5 demonstrates the narrative complexity of the age groups according to the means of the content scores within the episodes. The different colors in the bar diagram represent different episodes, and the width of the color in the bar represents the average content score of children in the age group in each episode. Notice that there are different numbers of content units in each episode. The episode structure and content units are presented in Figure 1 at the beginning of this article. The content scores presented in Figure 5 do not include the points received from answering the questions.

Out of the 4-year-olds (*N* = 9), five included the climax, two included the building of the climax, one included the result, one included the beginning, and no one included the action episode in their stories. The most complex story for a 4-year-old included the building of the climax, the climax, and the result. Three of the 4-year-olds did not include enough information for any of the episodes. Only the 4-year-olds excluded the action episode totally and mainly focused on the climax of the story. All the other age groups were likely to include all the episodes in their stories.

In the 5-year-old age group (*N* = 10), the mean result shows that at this age it can be expected for a child to be able to produce all the episodes in their story. However, when looking at the specific stories of the 5-year-olds, only two of them produced all the episodes. Most of the stories in this age group were missing either the setting statement or the result. For every episode, at least half of the 5-year-old included the episode in their stories (setting statement *N* = 6, action *N* = 6, building the climax *N* = 9, climax *N* = 9, result *N* = 5).

Based on the mean scores of the 6-year-olds (*N* = 10), story complexity seems to deteriorate. Of the 10 6-year-olds, only one included all the episodes in their stories. This child was the only 6-year-old who included the result episode in their story. Half of the 6-year-olds included a setting statement in their story, and more than half included the action (*N* = 6), building climax (*N* = 8), and climax (*N* = 6) in their stories. One of the 6-year-olds did not produce enough information for any of the episodes.

Among the 7-year-olds (*N* = 18), a total of five children did not produce enough information for any of the episodes. Of these five, four were able to answer the questions about the story, which indicates that they understood the story, but for some reason they did not produce stories themselves. One-third of the 7-year-olds produced all the episodes. A setting statement was found in 10 of the stories; action, climax building, and climax in 13 stories; and the result in 8 stories among the 7-year-olds.

Among the 8-year-olds (*N* = 12), five children produced all the episodes. All the 8-year-olds produced at least one episode. Almost all the 8-year-olds (*N* = 11) produced the action, building of the climax, and the climax in their stories. The setting statement was found in 8 stories, and the result was found in 5 stories.

Seven of the 9-year-olds (*N* = 13) produced all the episodes in their stories. One 9-year-old did not produce enough information for any of the episodes, but they were able to answer to all the questions about the story. All the others in this age group included the action and the climax building episodes in their stories. The climax was included in 11 stories, and the setting statement and the result were included in 9 stories.

Six of the 10-year-olds (*N* = 9) included all the episodes in their stories. All the 10-year-olds included the action and climax building episodes. The setting statement was excluded from one story, the climax from one story, and the result from two stories.

Eight of the 11-year-olds (*N* = 13) included all the episodes in their stories. There was one child who only included the setting statement in their story. All the other children included the action, climax building, and climax in their stories. The setting statement was excluded from one story and the result from five stories in this age group.

The results clearly demonstrate that the number of episodes in children’s produced narratives increases according to increasing age. Additionally, as can be seen from Figure 5, the number of content items inside each episode seems to increase with age as well. Already from the age of 5 years onward, there were children who produced all the episodes of the story, but the content of the different episodes was limited among children from younger age groups. The content of the different episodes among children under the age of 8 seemed limited compared to children 8 years old and older. Thus, there seemed to be a shift in narrative complexity among the children studied. The features in the story content and structure formed together the complexity of the story. The complexity of the narratives produced by children under the age of eight appears to be lower than the complexity of the narratives produced by children aged 8 and older.

## Discussion

### Development of narrative macrostructure among children acquiring FinSL

The current study focuses on the macrostructure of the narratives signed by children acquiring FinSL with the aim of providing insight into the development of narrative skills between ages 4 and 11. The aim of the first research question was to determine if there is a correlation between age and the production of narrative structure and content. The results show that the narrative structure episodes and the content units increased with age among the children studied. Additionally, when counting the total scores of the FinSL-PT, including the scores of story content, structure, and produced FinSL grammatical elements, the children’s results increased with age. These results are in line with studies conducted with other signed and spoken languages (e.g., Marschark et al., 1994; Morgan, 2005; Muñoz et al., 2003; Reilly, 2001; Schneider et al., 2006), and the narrative skills of children acquiring FinSL increased with increasing age.

The aim of the second research question was to determine what episodes of narrative children in different age groups produce and whether the complexity of the narrative increases with age. The results show that complexity and age have a strong correlation, as both content scores and structure scores increase with increasing age. In all age groups, the most likely episode to be missing was the result. Additionally, the setting statement was missing more often than the action, building climax, and climax episodes. All in all, among the older children, the probability for all the episodes to be included in the story was greater.

The 4-year-olds’ stories were still very limited, consisting mostly of the climax. This result was expected, as it has been shown that at this age children tend to focus on surprising events (Bruner, 1990). The results show a significant development in narrative skills between the ages of 4 and 5, which was also to be expected based on previous research findings (e.g., Muñoz et al., 2003; Price et al., 2006). There is inconsistency between ages 5 and 6. According to the results, there seems to be some kind of developmental gap in narrative complexity between 5-year-olds and 6-year-olds. The narrative complexity of 6-year-old children seems to be lower compared to 5-year-olds. The reason behind this result is not clear. When looking in relation to the result and individual factors, for example, hearing status, educational language, or the assessment situation done remotely or in person, a significant effect on the result was not found. In the 5- and 6-year-old age groups, there were only 10 children each. The main reason for this finding might relate to the rather limited amount of data inside each age group. This finding is also linked to what is known about language development at this age and that there might be great variation in narrative skills among children at this age. Even though at this age children are able to produce coherent stories with all the episodes, it is still typical that the references and the internal logic are somewhat unclear and that the recipient may find it difficult to understand the narrative (Loukusa et al., 2020). However, in the future, it is important to study the features of narrative development in more detail (e.g., the sign language grammar features) and to compare the effects of linguistic environment on these children’s results.

The 7-year-olds formed the largest age group in this study (*N* = 18). This age group demonstrated how the results in one age group can vary: 6 of the children were able to produce all the episodes, while at the same time 5 of the children did not produce enough information for any of the episodes. According to previous research, the narratives produced by 7-year-olds usually include clear structural elements and reflect story grammar (Suvanto & Mäkinen, 2011). Thus, the result of this study suggests that most of the 7-year-olds produced stories that met the age group’s expectations. However, almost a third of the narratives produced by children in this age group seemed to lag behind what could be expected from 7-year-olds. Still, these conclusions need to be made with great caution. The FinSL-PT has not yet been standardized, and the norms for each age group are still missing. Additionally, what needs to be highlighted here is the bi- and monolingual backgrounds of the children studied. All the children who participated in this study were acquiring FinSL in bi- or multilingual contexts. The focus of this study was only to research one of the children’s acquired languages. Thus, only part of children’s overall language development was within the scope of this study. The wide variation in the narratives produced by children might relate to the features of their bi- and multilingual language acquisition, and FinSL as assessed here might not be the dominant language of a given child. This may also cause variations between children’s scores. Previous studies have well revealed that children acquiring sign language have varying opportunities to use sign language in their daily lives and varying access to other signers (Kanto et al., 2013). The features of children’s linguistic environments that affect their narrative skills will be explored in more depth in future studies.

There is a developmental step between the results of the age groups of 7 and 8-year-olds: almost half of the 8-year-olds were able to include all the episodes in their stories, and none of the 8-year-olds failed to produce information for at least one episode. The results show that children’s narrative skills develop quite rapidly up to the age of 8, after which development seems to slow down. In the research of Schneider et al. (2006), the narrative development of children was shown to start slowing down by 7, 1 year earlier compared to the results of the present study.

The complexity of the stories seems to peak at the age of 10. This result, although unexpected, is also found in other studies (e.g., Justice et al., 2006; Kaderavek et al., 2004). These studies have found that there seems to be a peak in narrative skills at 10 years old, and after that, children’s scores may start to slightly decrease. It is not suggested that the skills take a step back but that there are other factors affecting the scores, for example, motivation to complete the tasks or a desire to complete tasks as quickly as possible.

In general, with some exceptions in the 7-year-olds, the results show that the narrative skills of children acquiring FinSL develop on the level of macrostructure by the same principles as what is found in other studies in spoken and signed languages. The results show that the narrative skills of children acquiring FinSL follow the established developmental trend.

### Methodological remarks and implications

When eliciting narratives, it is important to consider what kind of material is used. Reiterating a story just told to them can feel difficult for children. Children can feel pressure to remember the exact words and forms of the original story and resolve the situation by saying that they do not remember anything. It can also be confusing for children why they must tell a story that was just told to them. Narrative tasks are also sometimes too difficult for children who are used to being the recipient, not the narrator. If eliciting is done with pictures, the task requires the narrator to be able to produce a story with a logical timeline and cause-and-effect relations without an example (Suvanto & Mäkinen, 2011). Puupponen and Kanto (n.d.) compare FinSL-acquiring children’s narratives elicited with picture stories and video stories. They found that children used different ways of creating meanings depending on the material used for eliciting narratives. When telling a story based on pictures, the children used less constructed action, depiction in which a signer enacts the actions, feelings, thoughts, and utterances of discourse referents by using different parts of their bodies, rather than when telling a story based on a video. Additionally, Crawshaw et al. (2020) suggest that the cognitive processing of video materials is different from that of still images, which may benefit and support retellings.

The FinSL-PT has instructions for the assessor on how to build and carry out the test. The instructions ensure that the data are collected systematically and all the tests are comparable. In this study, if a test situation did not follow the instructions, the video was excluded from the data.

Because of the COVID-19 pandemic, some of the assessments were carried out remotely. In the remote assessments, the camera angles were different than in the other assessments. In most cases, the remotely recorded assessments were able to be annotated and scored. However, in some remote cases, a child’s signing was not completely visible in the recording, for the camera angle was narrower than in live situations. Although there were cases in which some of the signs produced by the child were not visible in the video, these signs were single signs that had no effect on understanding the narrative, and these cases were not excluded from the data.

It is important to be aware that children may perform differently if a task is carried out via video call compared to a live situation. Additionally, there might be interruptions in the child’s environment over which the researcher has no control, unlike in a situation in which the researcher is physically present. The pandemic made it difficult to conduct research in general on a global scale. The situation was also very challenging for children and families. From the viewpoint of the ongoing survey on studying children’s FinSL skills, the pandemic came at a very challenging time, but the researchers felt it was important that the research continue. From a research ethics point of view, special sensitivity had to be applied when meeting children and families. Data collection for the study was only continued if both parents and children gave their consent. Before proceeding with the data collection remotely, the researchers also prepared instructions on how to conduct the FinSL-PT tasks with the children via video call. This ensured that the data continued to be collected in a consistent and systematic manner. This experience eventually led to the construction of a set of practices that will allow the task to be carried out remotely even after the pandemic has ended. These practices will be described in more detail in a future publication.

Technical difficulties and remote connection lags in some cases caused short cut-offs in the recordings. These cases were not excluded if the cut-offs were so short that they did not interfere with the overall understanding of the narration. One case was excluded because of a cut-off that made it impossible to assess the setting statement and the action episode.

When assessing children’s language, the situation is always very sensitive, and it is not possible to rule out all the factors that could interfere with a child’s ability to complete the task at that specific moment. Considering the results in this study, it seems that the assessments done via remote connections were equally executed as were the assessments done in live situations. In the preliminary analysis of this study, there was no clear significance found in the children’s scores between assessments carried out remotely or in-person. However, in assessment situations, there is a difference between remote or in-person assessments, which might have effects on the child’s performance and the interaction between the assessor and the child, even if it does not clearly reflect in the child’s scores. In the future, the reliability of live and remote assessments and the effect the different assessment situations have on the results should be further investigated with more detail.

Because of these situational factors, the limitations of individual tasks and variations in children’s language skills should never be based on one task. Although narrative tasks are a sufficient way of assessing children’s language skills, they need to be supported by other tests to make reliable assessments. Several studies (Fey et al., 2004; Pankratz et al., 2007; Schneider et al., 2006) suggest that other measures of language skills are needed to conclude if the low scoring of narration tasks is only due to normal variations in children’s skills or if there is a possibility of challenges in language development. For FinSL and sign languages in general, there is a limited number of tools available for assessing the language development properly. It is important to continue to study the acquisition of sign languages and to develop assessment material designed especially for sign languages. Thus, in the future, it is also important to explore how different aspects of language skills, such as receptive and productive skills of vocabulary or grammatical structure, are linked to narrative skills.

The small size and heterogeneity of the study sample may make it difficult to draw reliable conclusions from these results. There are no statistics on children acquiring FinSL, but relative to the population of Finland (5.5 million), the data can be seen to be relatively extensive. The backgrounds of the children in the study differ greatly in terms of, for example, hearing status, hearing age, exposure to sign language, and languages used at home, day care, and school. However, the heterogeneity of the children acquiring sign language is a reality, and to obtain a realistic picture of the language development in this group, the whole group must be studied.

## Conclusion

There have been very few studies about the language development of children acquiring FinSL. This study is the first step in creating a reference value for assessing narrative macrostructure skills in FinSL. The aim was to include children with different backgrounds, language environments, and hearing status to obtain results that reflect the diversity of the group as well as possible.

In this article, we have not analyzed the microlevel of the narratives nor the possible impact of children’s hearing status, language environment, or other background factors on their narrative skills. In both areas, the data consist of multiple factors that will be analyzed in more detail in later studies. Although in the sign language field it is customary to take hearing status into consideration when analyzing research results, in the preliminary analysis of this study, the role of hearing status in the results was not clear enough to be included in this sub-study analysis without researching other factors in language environments more thoroughly. FinSL-PT allows to analyze the child’s ability to produce sign language from many different grammatical aspects, and to be able to concentrate on every aspect, we have excluded the microstructure of the narratives from this article, and it will be discussed in detail in a future article.

For the narratives in this study, the children used a variety of methods to create meaning, including signing, acting, fingerspelling, descripting, depicting, and gesturing. All these means of expression were accepted, and the children were each given a content point if the assessor was able to understand what the child meant. This multimodal approach to narratives is important in sign languages, as sign languages are hybrid systems in which conventionalized language and gestures interact on a continuum. When research has been conducted on adults signing narratives, the results show that individual differences in how to tell or show the actions in a story vary, and there is no one correct way to produce a narrative (Jantunen, 2017). This approach to analyzing the narratives in sign languages is important because of the nature of sign languages, and it also serves another function. Nonverbal communication is part of all human interaction, including in spoken languages, and it is especially emphasized in children’s interactions (Tykkyläinen, 2011). Even though we are aiming to conduct a future study focusing more closely on the microstructure of the children’s stories, we argue that by focusing first solely on the macrostructure, we can differentiate a child’s ability to correctly use one specific language from their ability to understand and produce understandable narratives. This distinction may allow us to better understand the reasons behind possible indications of atypical language development.
